# Design and development of a low-cost glazing measurement system

**DOI:** 10.1016/j.mex.2020.101028

**Published:** 2020-08-14

**Authors:** Yanxiao Feng, Julian Wang

**Affiliations:** Department of Architectural Engineering, Pennsylvania State University, University Park, PA 16802, USA

**Keywords:** Glazing systems, In situ measurement, Sensor, Energy efficiency

## Abstract

Knowledge of the properties and performance level of a glazing or translucent component system is necessary for assessing the operational energy use of building or greenhouse structures and proposing potential strategies for saving energy. The study of in situ measurements of the thermal and optical performance of glazing systems has not yet been thoroughly examined. To accomplish this task, a portable and easy-to-use in situ measurement system for key glazing properties that uses the inexpensive Arduino platform and compatible sensors was designed, 3D-printed, and fabricated. The glazing properties that can now be measured include the center-of-glass U-factor, solar transmittance, and visible transmittance. In this method-focused paper, we present the step-by-step development procedure in detail, as well as describe the necessary hardware and codes. One can use the shared files to 3D-print the sensor cases and follow the described procedure to achieve the measuring system hardware. Subsequently, the shared codes, including the calibration functions for the associated hardware and the “self-examination” procedure can be applied to achieve a working system for measurement. In brief, this new method is:

• Based on thermodynamic equations and easy-to-use.
• Open source, low cost, and simple to assemble.
• Able to automatically determine the effectiveness of the output via the developed diagnostic algorithms.

## Graphical abstract

**Figure:**
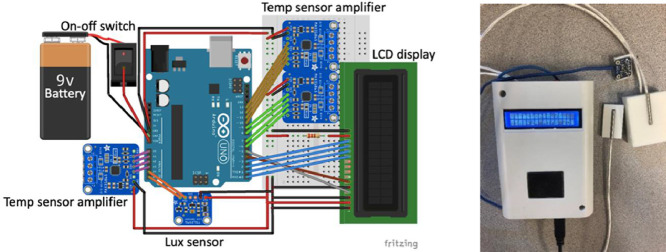

Specifications table

**Table:** 

Subject Area:	Engineering
More specific subject area:	Architectural Engineering
Method name:	Arduino-based non-destructive in situ glazing measurement
Name and reference of original method:	*N/A*
Resource availability:	*All available resources have been included in the text and the online data repository (in the supplementary material section)*

## Method details

The glazed window system, as an essential component of the façade, can significantly affect the performance of the lighting, heating, and ventilation in buildings, thereby determining the building energy usage and the indoor user comfort [1]. The National Fenestration Rating Council (NFRC) has established a reliable and widely used energy performance rating system for building windows in North America, in which the primary building window properties include Center-of-glass U-factor, Solar Heat Gain Coefficient (SHGC), and Visible Transmittance (VT). Measuring and knowing these window properties of existing buildings would become an essential step for evaluating their energy performance levels and making decisions of window retrofitting, replacements, or upgrades. For instance, the ratio between VT and SHGC has been deemed as a key indicator for selecting the ideal window product in hot climates [2,3].

In particular, for the physical measurement of the Center-of-glass U-factor, according to the standardized test procedures defined in ASTM C1363 [4] or ISO 15099 [5], it is achieved by measuring the heat flow transmitted through the glass and the internal and external windowpane temperature difference within certain environmental conditions in terms of temperature, solar irradiance, airflow speed, etc. Following this method, several in situ U-factor measurement tools have been developed by using heat flux sensors and surface temperature sensors, such as the gOMS of greenTEG [6], the TRSYS of Hukseflux [7]. Regarding the measurement of SHGC, the calorimetry hot box method specified in NFRC 201 [8] as well as the method defined in ISO 19467 [9], typically require instruments including solar calorimeter, pyranometers, temperature sensors, and flowmeters. In addition, the VT measurement procedure for window product test samples defined in NFRC 200 [10] and ISO 15099 [5] relies on specific test apparatus, photometric sensors, and stable atmospheric conditions. The challenge in the current development of in situ window properties measurement is three-fold. First, due to the strict requirements on measuring environment and surrounding circumstances, in situ measurement procedures have not been specified in current standards and guides. Second, although there are some professional instruments available in practice, the tools are either costly or over-complicated to be used on site. Last, no such multifunctional system measuring both thermal and optical window properties has been developed until now.

To address these issues, in this work, the Arduino microcontroller board and diverse low-cost sensors were used to capture the signals and interact with building indoor and outdoor environments. A simplified but scalable in situ measurement system that enabled the combined measurements of the properties mentioned above was developed, in which the thermal conductance calculation was not relying on heat flux sensors but rather achieved by using a pre-designed 3D printing block and simple temperature sensors. Furthermore, a self-examination procedure was embedded to aid the effectiveness determination of measuring environmental conditions. In particular, it used electronic elements that were inexpensive and small in size, including a luminosity sensor, surface temperature sensor, 3D-printed acrylonitrile butadiene styrene (ABS) block, and display screen. The finished module employed a user-friendly interface with a display screen that shows the data measured in real-time. The major window glazing properties, including center-of-glass U-factor, solar transmittance (τ_s_), and visible transmittance (VT), were measured based on the governing equations. The digital lighting sensor was calibrated and used to measure light irradiance and illuminance on a glass surface via τ_s_ and VT calculations, respectively. The surface temperature sensors combined with the 3D-printed ABS blocks were intended to measure the parameters necessary to calculate the center-of-glass U-factor. Some of this research method borrowed the heat flow meter method defined in the ISO 9869 standard [11], while the unique features of the proposed method (as compared with other in situ measurements) were the focus of transparent building elements, the combined measurement of center-of-glass U-factor, solar transmittance, and VT, and the ability to apply the thermal coefficient measurement method without the need for relatively expensive heat flux sensors.

## Materials and tools

• Arduino Uno (https://www.arduino.cc/en/Guide/ArduinoUno)
• Light sensor (https://www.adafruit.com/product/1980)
• Three platinum RTD sensors (https://www.adafruit.com/product/3290)
• Three thermocouple amplifiers (https://www.adafruit.com/product/269)
• On-off power switch
• Protoboard
• 9v battery
• LCD display
• Flexible copper wires
• Header pins
• Electric insulation heat shrink tube
• Resistor
• 3D printer
• ABS material
• Soldering pen kit

## Key sensors setup

1) Lighting sensor setup

The TSL2591 digital luminosity sensor was selected because it can be used in a wide range of light conditions. The lux range it measures is from 188 µLux to 120,000 lux. It is also more accurate than other cadmium sulfide cells and enables exact lux calculations both in infrared and full-spectrum diodes [12]. The lux sensor was used to measure two wavelength ranges of solar radiation: the visible light range and full-spectrum light range for VT and solar transmittance calculations, respectively. Two professional instruments were used to generate the reference values needed to calibrate the lighting sensor (see Table 1).

**Table 1:** Professional instruments used to calibrate the simplified sensing module outputs.

Sensor	Output (unit)	Professional Instrument
Adafruit TSL2591	Spectral irradiance (w/m^2^)	ASEQ LR1-T v.2
	Visible light illuminance (lux)	KMT-10MA

**Fig. 1:**
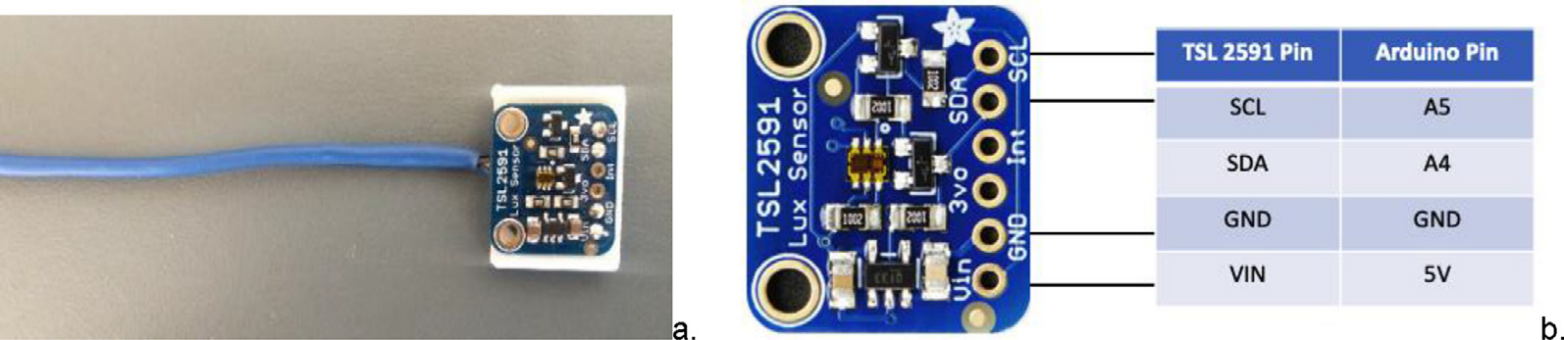
(a) Lux sensor processing and (b) TSL2591 wiring.

**Fig. 2:**
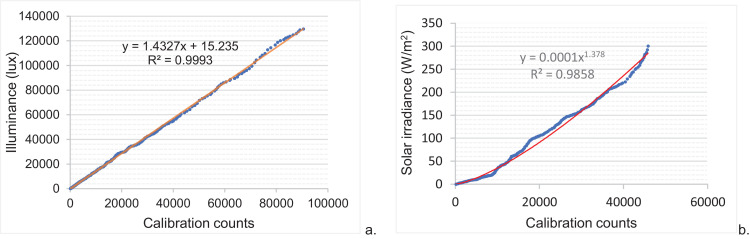
Correlation between the sensor and meter measurements: (a) illuminance calibration and (b) spectral irradiance calibration.

The lux sensor was connected to four wires that were wrapped in extended insulation plastic. These were enabled by placing the light sensor a certain distance away from the user (see Fig. 1a). The sensor communicated with the Arduino board by sticking to the A4 and A5 analog input pins (see Fig. 1b). For visible light, the TSL2591 measurement range was from 0 to 120,000 lux. The getLuminosity function was used to read the raw data from the visible spectrum light sensor, and correlations were made with the illuminance values obtained from the KMT-10MA. There were 300 lighting datasets gathered. The regression line for the wide lux wavelength range is shown in Fig. 2a. This regression function was written in the Arduino code to directly output the accurate lighting level.

For the full spectrum of solar irradiance, a power relationship was found between the source signal for the full spectrum from the TSL2591 light sensor and the energy measured in LR1-T (see Fig. 2b). Similarly, the getFullLuminosity function read the full spectrum sensors and returned the values, which were then compared with the irradiation measurements via the ASEQ LR1-T v.2 spectrometer. The source signal for the full spectrum could have been as high as 50,000, approximately indicating the solar irradiation on a sunny day at 2:00 PM in June in Cincinnati, Ohio. The power function could also be put in the Arduino code and irradiation directly read in the LCD display.

2) Surface temperature sensor setup

**Fig. 3:**
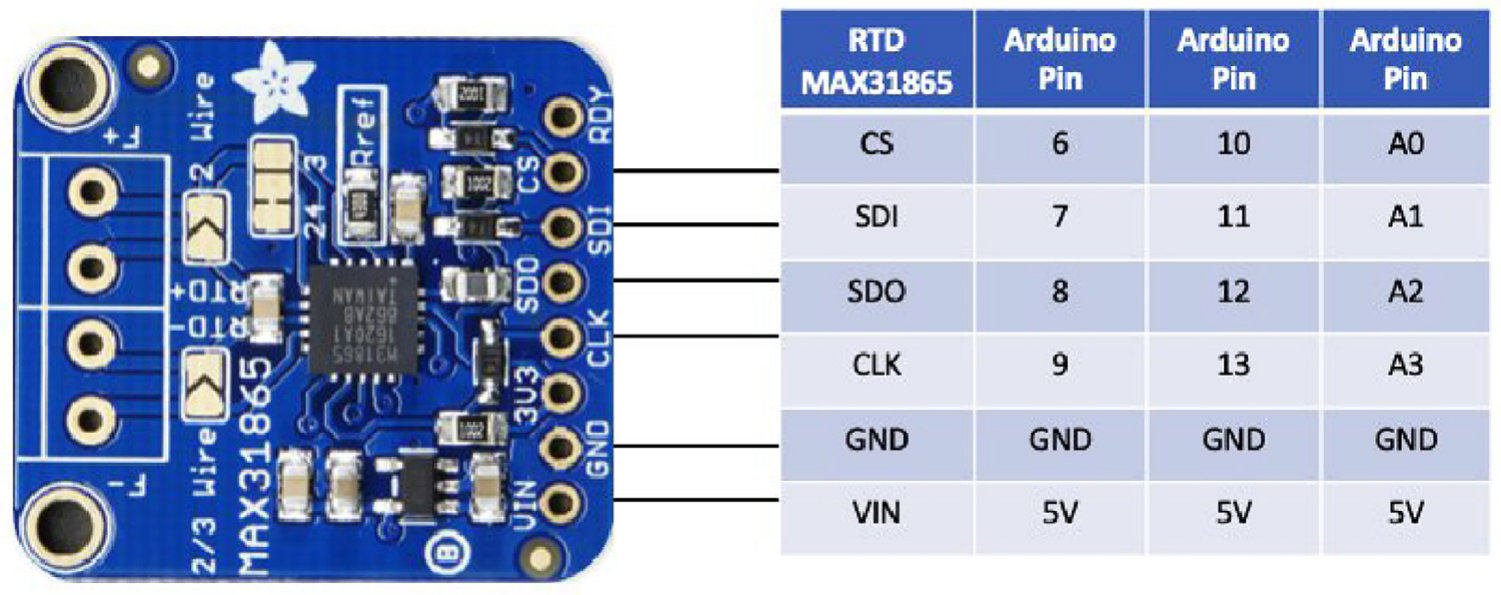
3 Max31865 wiring with Arduino pins.

**Fig. 4:**
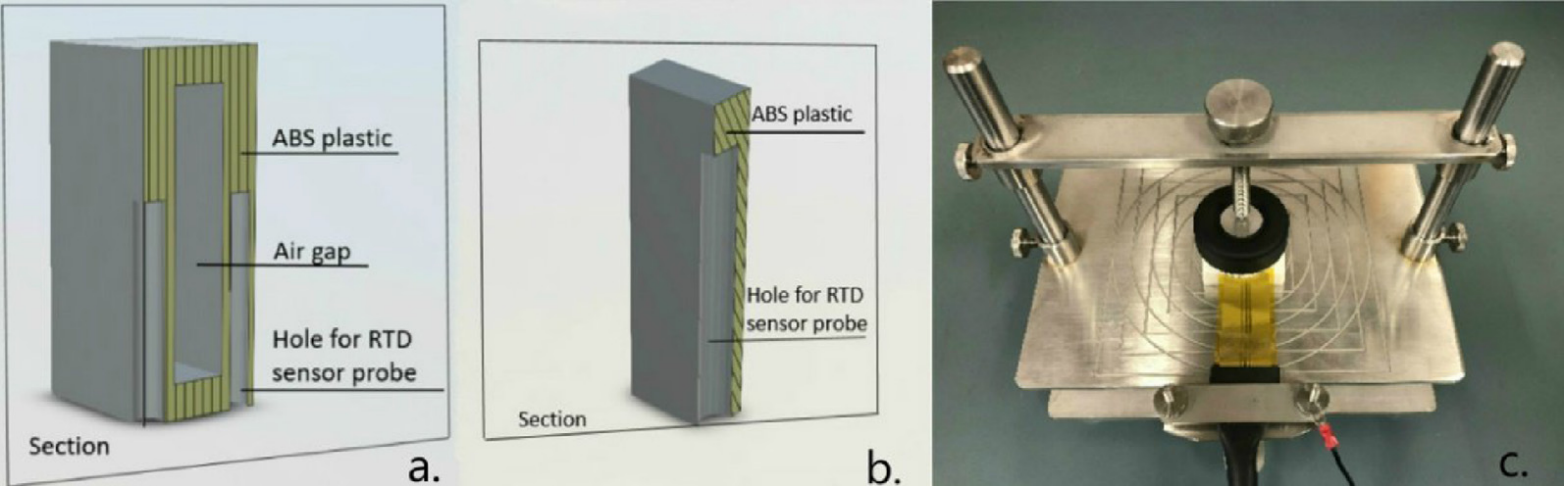
Section view of cuboid parts: (a) indoor module, (b) outdoor module, and (c) thermal resistance measurement.

**Fig. 5:**
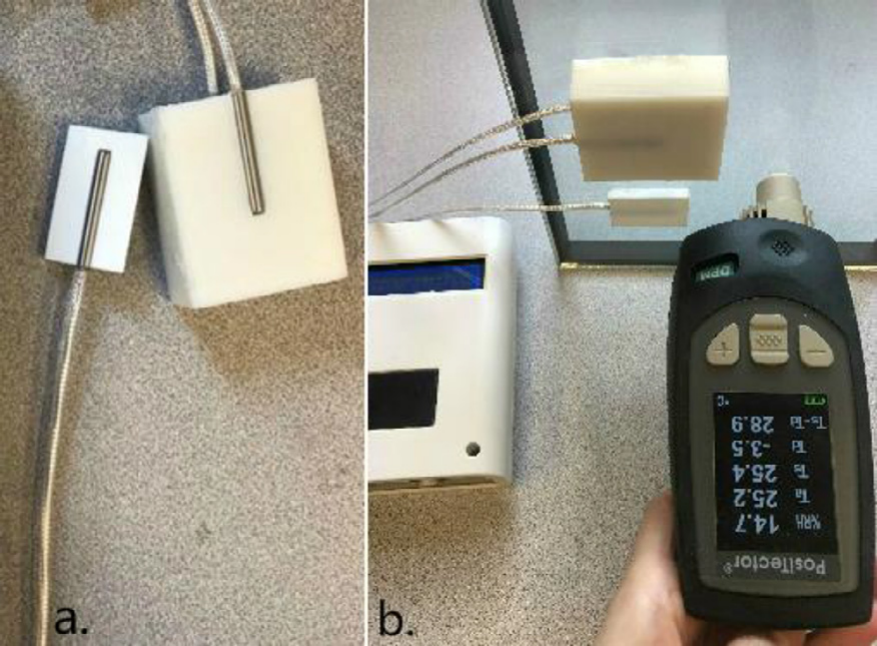
(a) 3D-printed cube with embedded sensor probes, and (b) surface temperature sensor calibration using the PosiTector.

The temperature sensor amplifier required four input pins to communicate with Arduino. The three sets of analog pins connected to the sensors were analog pins A0 to A4 and digital pins 6–9 and 10–13 (see Fig. 3). The temperature sensors were then connected to the amplifier to allow the measurements to work. Regarding the property center-of-glass U-factor, we used two 3D-printed objects made by ABS. Sectional views of the interiors of the two cuboids are shown in Figs. 4a and 4b. There was an air gap inside each of these objects to increase their thermal resistance and obtain more distinct surface temperature values from each side. Different sizes of the block were tested based on the requirement that the length was longer than that of the embedded temperature sensor tube and the thickness just sufficient to include two sensor probes and an air gap. In this project, the dimensions of the indoor module were 40 × 19 × 50 mm; the outdoor module dimensions were 5 × 20 × 35 mm. The thermal conductivity of the 3D-printed cuboid was measured using the hot disk measurement method and the TPS-M1 system from Thermophysical Instruments (see Fig. 4c). The thermal resistance of the 3D-printed object was 0.37 m^2^•K/W. The diameters of the holes for the RTD sensor probe were slightly larger than those of the probes to force the probes to squeeze into the holes and stay tightly in place. The intersection of the cylindrical hole facet in the outdoor module and surface of the cube was almost a tangent line, enabling the sensor probe to firmly come in contact with the glass surface. One of the holes in the indoor module had the same position, the almost tangent location towards the outside direction; the other hole was in the almost tangent location towards the inside direction (see Fig. 5a). The temperature sensor probe was calibrated using PosiTector, as shown in Fig. 5b. The difference between the two values was found to be minimal.

*LCD display screen and enclosure*

**Fig. 6:**
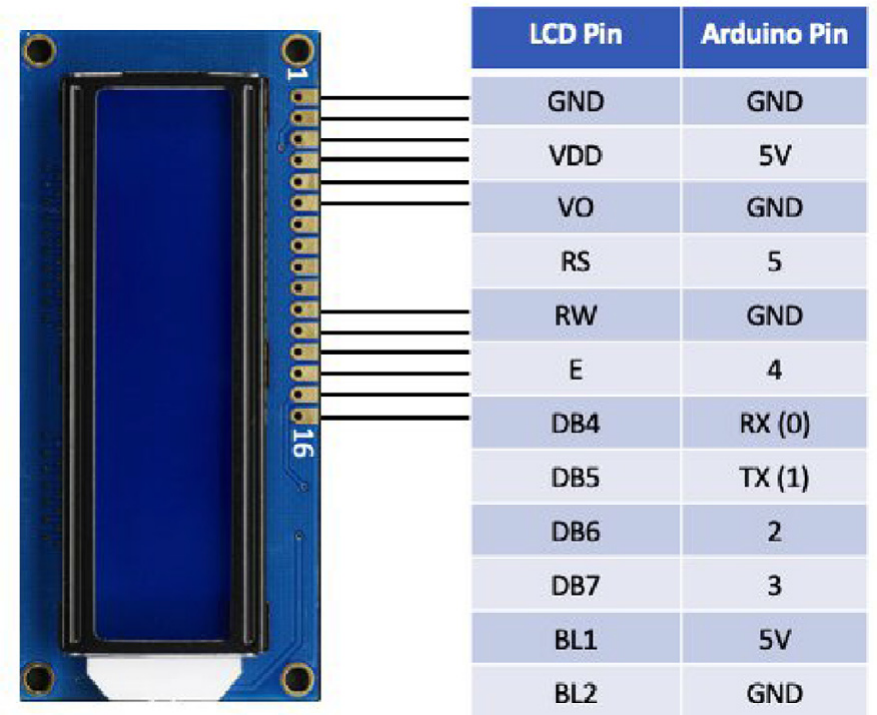
LCD display wiring.

A standard LCD screen was selected to display the real-time data collected using the Arduino module. The LCD screen was 16 characters wide and had two rows. The LED backlight could be dimmed with a resistor. It required six input pins to communicate with the Arduino board: serial out (TX), serial in (RX), and digital pins 2–5 (see Fig. 6). The enclosure case to hold and protect the electric components could be 3D-printed or bought online. However, this enclosure needed to have built-in space to accommodate the shapes of different components.

3) Wiring and assembly of all components

**Fig. 7:**
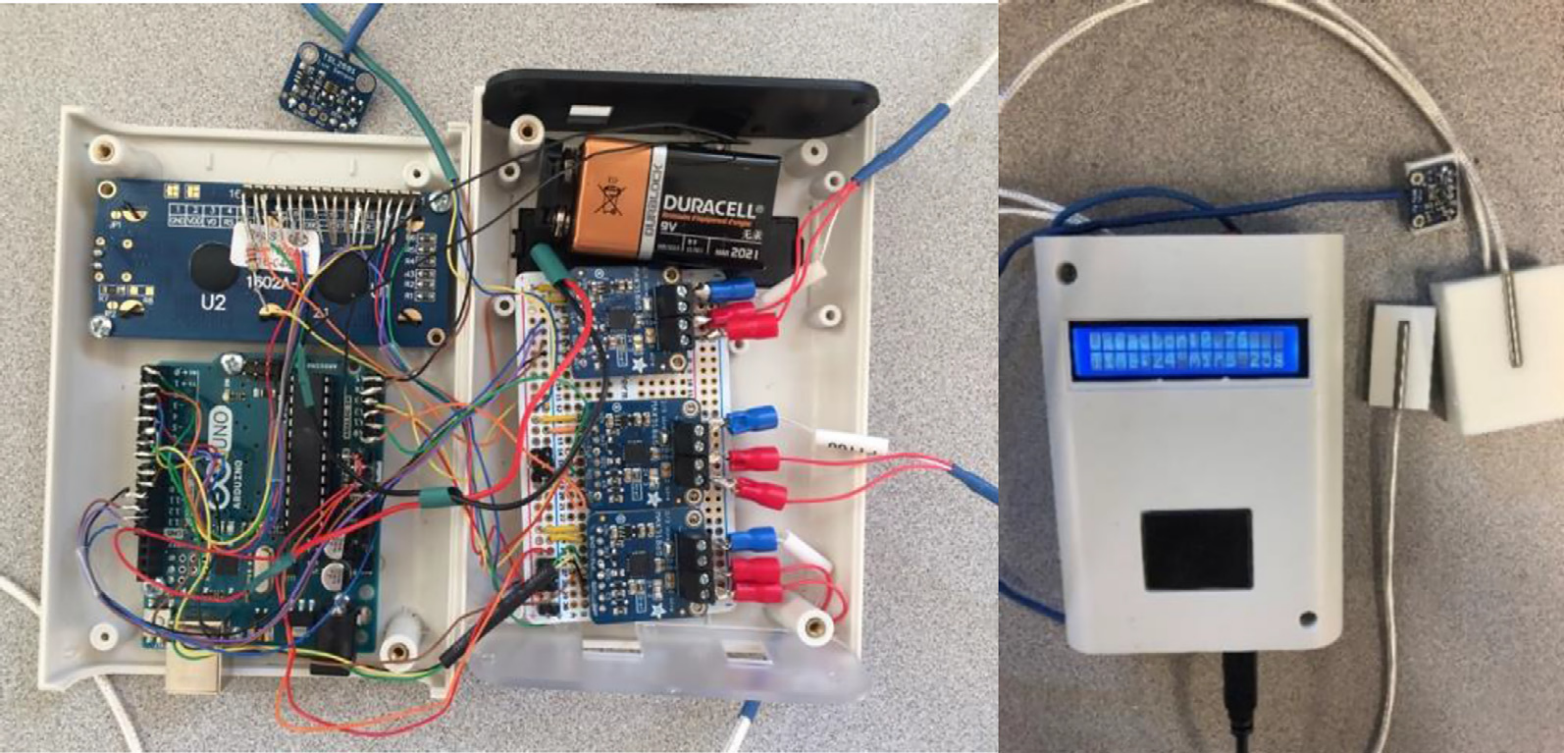
(a) Interior placement and (b) fabricated instrument.

All of the electric parts were placed in an Arduino enclosure, including the LCD display, battery, holes for USB connection, and on-off button. The interior connection and finished look of the final fabricated instrument are shown in Figs. 7a and 7b. To make this module more compact and portable, a protoboard was used to connect the sensors’ power and ground pins. Each hole of the board was surrounded by a copper pad to enable connections by either bridge soldering or running wires. The enclosure fit the Arduino and standard 16 × 2 LCDs; screws were used to fasten the components.

## U-factor measuring module mechanism and design

**Fig. 8:**
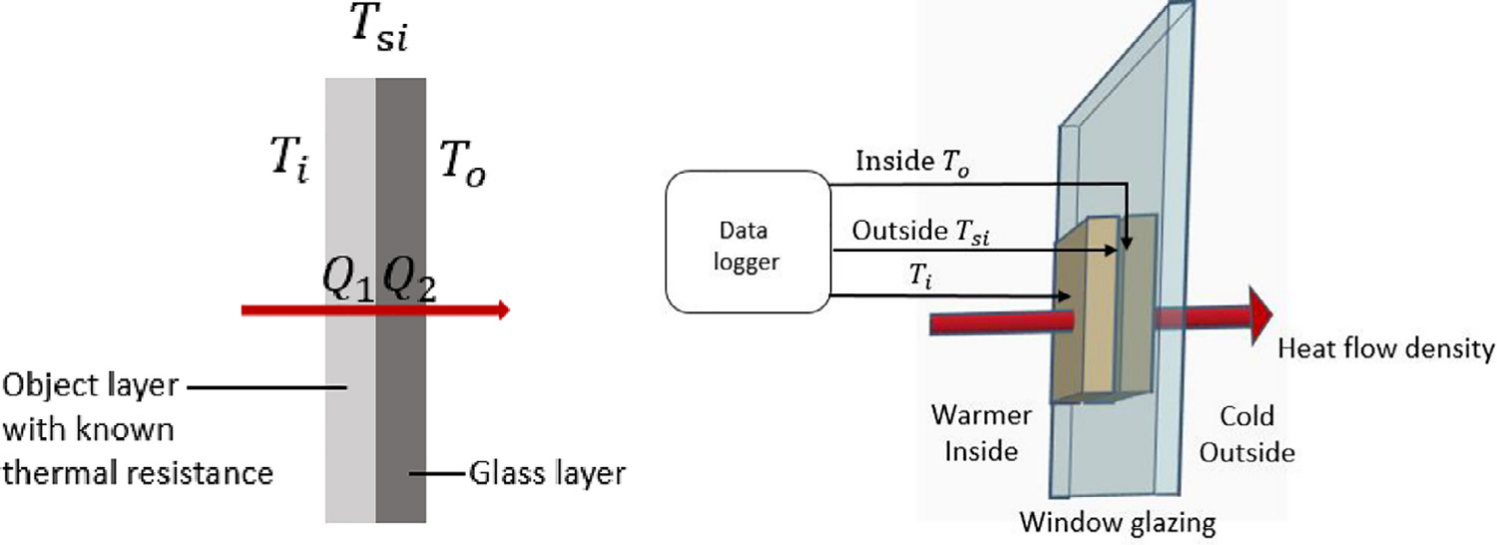
(a) Heat transfer through multi-layer surfaces, and (b) typical measurement setup.

In order to measure the heat flux through the glass, the temperatures of the different surfaces of thick and thin cuboid surfaces needed to be measured. When the width and height of insulation are far greater than its thickness, it can be assumed that the heat flux intensity is equal throughout the insulation. Thus, it was concluded that the heat flux intensity through the cuboid was equal to that traveling through the glass. The underlying mechanism is explained in Fig. 8a. in a steady state, the heat flux intensity is equal in the different layers: Q_1_ = Q_2_. This is assuming that Q_2_ is through the glass layer with an unknown U-factor and Q_1_ is through another object with a known level of thermal resistance. Consequently, we obtained Q1 using Eq. 1. Then, the U-factor was obtained using Eqs. (2) and (3).

(1) $$Q1=\frac{1}{R}*A*\left(T_{i}-T_{si}\right)$$

(2) $$Q2=U*A*\left(T_{si}-T_{o}\right)$$

(3) $$U=\frac{T_{i}-T_{si}}{(T_{si}-T_{o})*R}$$

where U is the center-of-glass U-factor of the glass (W/m^2^K), T_i_ is the temperature of the object's surface exposed to air inside the glass (in °C), T_si_ is the temperature of the interface between the object and glass (in °C), T_o_ is the outside glass surface temperature (in °C), and R is the thermal resistance of the 3D printed object (in m^2^·K/W).

A typical measurement setup can be seen in Fig. 8b. The indoor object consisted of two surface temperature sensors. One was embedded in the surface that stuck to the glass, and the other one was embedded in the opposite surface exposed to the air during the actual in situ measurements. The outdoor object was stuck to the exterior surface of the glass to measure the exterior glass surface temperature. With these two 3D-printed objects, we obtained three surface temperatures: T_0_, T_i_, and T_si_. The 3D drawing files used for the 3D-printing process can be found in the supplementary material section. These three measurements were received and processed every second by the microcontroller. By employing Eqs. 1 through 3 with the known insulation of the printed object, the U-value could then be read through the LCD display. A quasi-steady state was assumed for this measurement system when the “self-examination” procedure was finished and the U-factor output. First, the overall sensing and measuring system required 30 minutes to be stable. Afterwards, the variation in the U-factor calculated between the average and maximum in a five-minute period was smaller than 5%. Then, the so-called quasi-steady state was assumed to be reached and the average U-factor output via the LCD screen. The detailed explanation of the quasi-steady state and the designed “self-examination” procedure can be found in our previous paper [13].

## VT and **τ**_***s***_ measuring module mechanism and design

The NFRC's VT rating was for the whole window area, including the frame and grid spaces that do not transmit any light; therefore, the VT for the glass was higher than the NFRC's VT. Since glass is the main contributor to a window's VT, the VT measurement facilitated by this design enables homeowners to determine the passive lighting condition of their homes. Visible light transmittance is the percentage of visible light transmitted through a window. The equation that describes VT is:

(4) $$\text{VT}=\frac{L}{L_{T}}$$

where L is the daylight passing through the glazing and L_T_ is the total daylight landing on the glazing.

*τ_s_* represents the direct solar transmittance through a window. In measurements made via the current design, the incident and transmitted solar irradiances should be measured at the same time and location to reduce errors attributable to changes in solar irradiance. The solar transmittance *τ_s_* can be calculated from Eq. (5):

(5) $$\tau_{s}=\frac{E}{E_{T}}$$

where E is the solar irradiance passing through the glazing and E_T_ is the total solar irradiance landing on the glazing.

For the properties of VT and solar transmittance measurement, the procedure defined by the NFRC and ISO needs specific apparatus, photometric sensors, and pyranometer sensors. However, neither NFRC nor ISO standards define the in situ measurement procedure. The tool developed in this work adopted one light sensor consisting of two light-responding photodiodes (visible plus infrared). Two integrating analog-to-digital converters could transfer the photodiode currents into a digital output, representing the light irradiance measured on each photodiode. Then, the original irradiance was used to compute the solar transmittance, τ_s, and the processed illuminance in lux derived by applying the photopic luminosity was used to calculate the visible transmittance, VT. The primary difference compared with the specified procedure in the standards is the design with the coupled (visible and infrared) lighting sensor, so it could output both radiometric and photometric quantities for calculating visible and solar transmittances, respectively. Therefore, comparatively, the method designed here could facilitate a more convenient operation and rapid estimation of the properties of the installed window systems.

## Implementation protocol

**Fig. 9:**
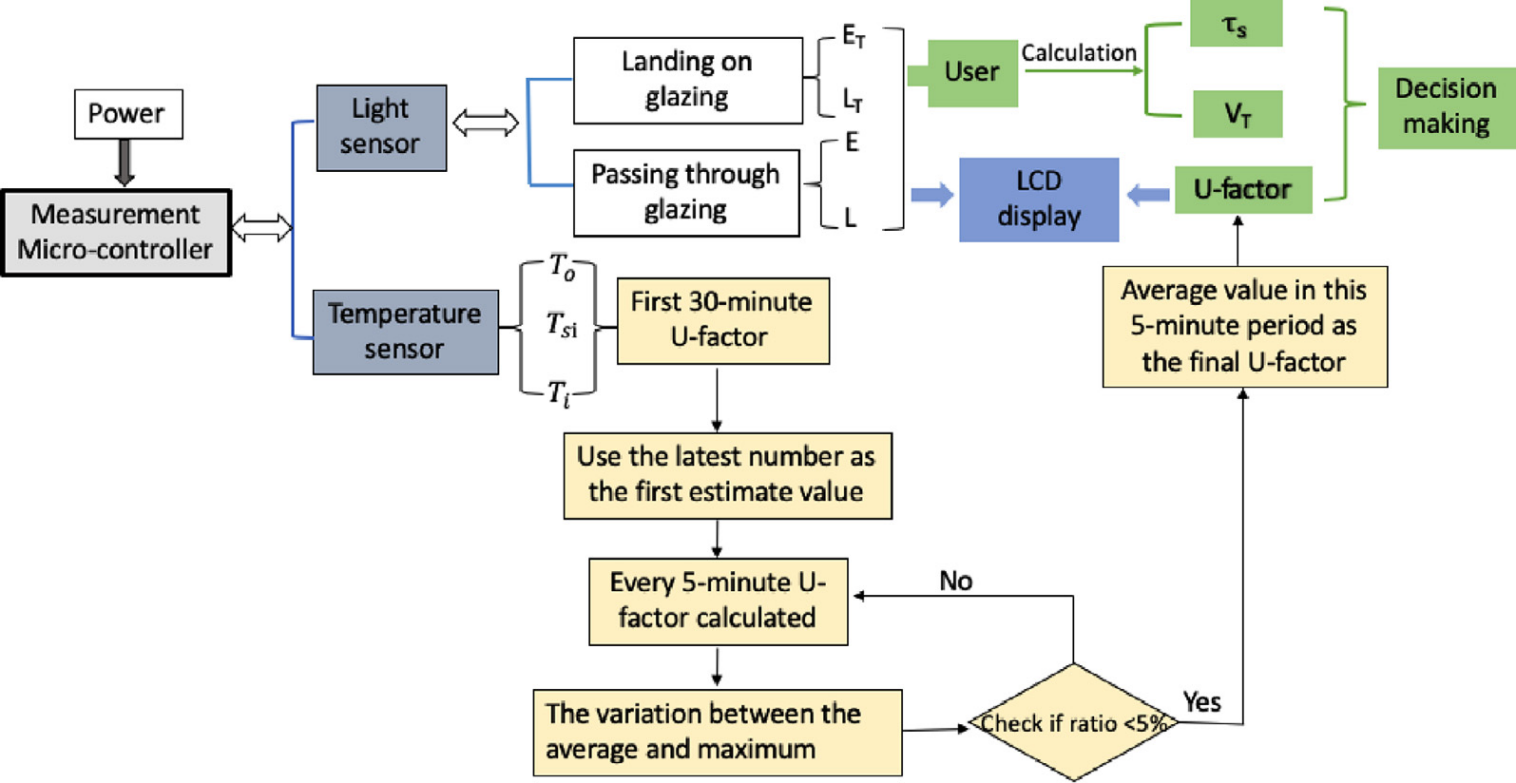
Measurement procedure.

All of the above-mentioned functions and equations were incorporated into the system via Arduino programming. The link to all the Arduino codes, including the calibration functions for the associated hardware items and the “self-examination” procedure, can be found in the supplementary materials section. One can follow the method described above and the shared 3D-printing files for the sensor modules to assemble the hardware and then apply the shared Arduino codes to achieve a working system for measurement. The measurement procedures for the three properties, including the “self-examination” procedure for U-factor measurement, are shown in Fig. 9.

## Validation

Three reference glazing samples, including clear double-pane, double-pane coated with hard low-E, and double-pane coated with triple silver low-E were used to evaluate the validity and reliability of the measured properties. Three experiments conducted during the early morning were set up with different solar radiation intensities to ensure the validity and reliability of the measured data. The results show that the error percentage for VT measurement was 2.8% on average, and the τ_s_ measurement was approximately 9% compared with the known values labeled in the three reference glazing samples. The metrics for the lighting and radiation measurements were obtained and calculated normally. The TSL2591 digital luminosity was cosine-correlated for broadband white light sources [14], and thus the measurements’ accuracy of radiation from all incident angles was guaranteed. The TSL2591 sensor would have been saturated under outdoor conditions with strong sunshine. Thus, placement angles and positions relative to the incident light substantially influence accuracy, especially in situations where more complicated and advanced coatings were used. For accuracy in the U-factor measurement, three measurement experiments on a double-pane window (two 6mm clear glass panes with an air gap) in a campus office building were configured and performed. The accuracy of the measurement system ranged between 6.1% and 21.6%, and the average error percentage for the measurements was strictly in line with ISO9869 was 7.95%. The measurement of the U-factor should be in line with ISO 9869 and avoid direct sun radiation and the effects of wind.

## Conclusion

In this work, an inexpensive and easy-to-use measurement approach and instrument was proposed and designed to enable home window measurements. One can easily program Arduino sensors and systems with sufficient open sources. In this project, 3D printing was used to fabricate the main instrument structure and cases, allowing the selected sensors and controller platform to be well-embedded. This enabled the approximate measurement of center-of-glass U-factor, solar transmittance *τ_s_*, and VT. Compared to the outcomes of professorial instruments, the results of this measurement method and tool offer quite a high level of accuracy under appropriate weather conditions. For the Center-of-glass U-factor measurement, an automated determination algorithm was particularly developed to evaluate whether the quasi-steady-state was achieved. The experiment results present that using this tool within the conditions with the indoor-outdoor temperature difference of at least 15 °C. Regarding the τ_s_ and VT parameters, even though the TSL2591 sensor has its cosine-corrected for white light sources [14], the travel length of the transmitted light would still be affected by the incident angle because of the existence of the glazing layer(s). Therefore, users need to observe the geometric relationship between the window and the solar position to avoid the potential errors by the oblique incident angles.

## Declaration of Competing Interest

The authors declare that they have no known competing financial interests or personal relationships that could have appeared to influence the work reported in this paper.
